# A Genetically Modified Skin Graft for Treating Alcohol Use Disorder and/or Polysubstance Abuse With Cocaine

**DOI:** 10.3389/adar.2021.10007

**Published:** 2021-06-18

**Authors:** Qingyao Kong, Xiaoyang Wu, Ming Xu

**Affiliations:** ^1^ Department of Anesthesia and Critical Care, The University of Chicago, Chicago, IL, United States; ^2^ Ben May Department for Cancer Research, The University of Chicago, Chicago, IL, United States

**Keywords:** skin graft, gene therapy, alcohol use disorder, cocaine abuse, co-abuse

## Abstract

Alcohol use disorder (AUD) is one of the foremost public health problems. Alcohol is also frequently co-abused with cocaine. There is a huge unmet need for the treatment of AUD and/or cocaine co-abuse. We have developed and used a skin stem cell-based gene delivery platform and found that production of the glucagon-like peptide-1 (GLP1) from the grafted genetically modified skin reduced development and reinstatement of alcohol-induced drug-taking and seeking, voluntary oral alcohol consumption and alcohol-induced increase in dopamine (DA) levels in the nucleus accumbens (NAc). Moreover, we have developed a novel co-grafting procedure for both modified human butyrylcholinesterase (hBChE)- and GLP1-expressing cells. Skin grafts-derived hBChE and GLP1 reduced acquisition of drug-taking and toxicity induced by concurrent alcohol and cocaine injections. These results imply that gene delivery through skin transplants may add a new option to treat drug abuse and co-abuse.

## Introduction

Alcohol use disorder (AUD) involves problems controlling drinking, continuing to use alcohol even when it causes problems, having to drink more to get the same effect, or having withdrawal symptoms when one decreases or stops drinking [1,2]. AUD is one of the most prevalent psychiatric disorders worldwide. About 18 million adult Americans have AUD [3]. AUD can change how brain functions and damage other organs. It can also increase the risk of death from driving under the influence, injuries, suicide and homicide. Excessive alcohol use is among the leading causes of preventable death [4]. There are three FDA-approved medications, disulfiram, naltrexone, and acamprosate, and behavioral counseling for stopping or reducing drinking and preventing relapse in humans [5]. However, only 1.3 million receive treatment [6]. Moreover, not all people respond to these medications and types of treatment, and compliance varies among those who receive treatment regimens. Significantly, AUD is also characterized by high comorbidity such as with cocaine. Cocaine is a widely abused drug that causes significant morbidity and mortality. From 2012 through 2018, the rate of cocaine-related overdose deaths more than tripled [7]. Although a variety of pharmacological targets and behavioral interventions have been explored, there are no FDA-approved medications for reducing cocaine use or treating relapse in cocaine addicts. Whereas there is a huge unmet need to develop treatment for cocaine abuse, the lifetime prevalence of occurrence of comorbid alcoholism in cocaine abusers is 50–80% [8,9]. Concurrent use of alcohol and cocaine produces cocaethylene which can inhibit DA transporters and elicits euphoria [10], and it has significant cardiotoxic effects by blocking sodium channels with a potency that is equal to or greater than cocaine [11,12]. Cocaethylene also has a longer half-life in the plasma than that of cocaine and the LD50 of cocaethylene is substantially lower than cocaine. As a result, there is a 20-fold higher risk of death than using cocaine alone [13,14]. Now, in *Molecular Psychiatry* [15], we reported that one skin stem cell-based gene delivery platform for treating AUD and/or polysubstance abuse with cocaine. This work expands the application of the cutaneous gene delivery platform for treating cocaine abuse and overdose related deaths [16–18] to additional drug abuse and co-abuse.

GLP1 is a gut neuropeptide hormone mainly secreted by intestinal enteroendocrine L cells and neurons in the nucleus of the solitary tract. These cells secret many additional hormones and peptides, including peptide YY, cholecystokinin, ghrelin, and pancreatic polypeptide [19]. These peptides play vital roles in glucose homeostasis, appetite, satiety and onset of obesity and type 2 diabetes via acting on gut-brain-axis. GLP1 receptors (GLP1R) are also distributed in brain reward circuits comprised of the ventral tegmental area and the NAc. Natural rewards can trigger DA surges in the NAc [20,21], and GLP1 or GLP1 analogs can suppress mesolimbic DA transmission or DA signaling in response to food-predictive cues and restrain palatable food intake [22,23]. Emerging evidence suggest that excessive eating, obesity and substance abuse share some of the neurobiological mechanisms involving the DA system [24]. For example, GLP1, GLP1R agonists and antagonists can modulate drug reward behaviors including those induced by alcohol and cocaine [25–30]. Moreover, *via* acting on the GLP1Rs in reward circuits, GLP1 can attenuate drug-induced neurobiological effects in mice including reward-seeking behavior and DA release in the NAc [28,29]. Since the safety profile of GLP1 analog drugs has been proven in the treatment of type 2 diabetes and obesity in human patients, its effectiveness in treating other diseases or conditions such as AUD will be highly favorable in new drug development. However, GLP1 has a very short half-life *in vivo* [31], and clinical administration of GLP1R agonists may be inconvenient and costly because it requires long-term and parenteral administration, which limits its application in treating AUD.

**FIGURE 1:**
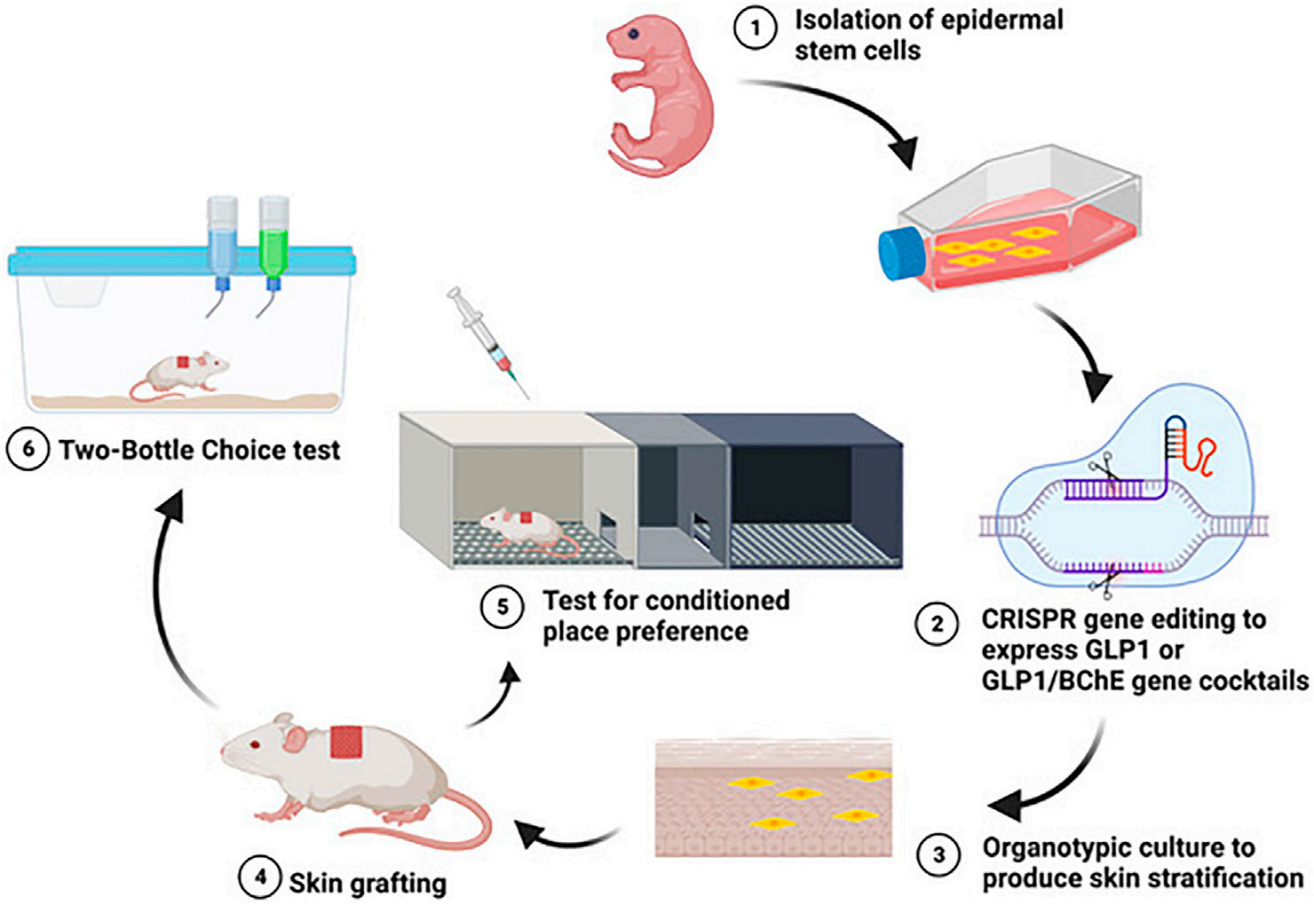
Using a skin cell-based gene delivery platform to reduce alcohol and/or cocaine abuse.

We previously developed a skin stem cell-based long-term gene delivery approach in mouse models that enables us to genetically engineer the skin stem cells with the CRISPR/CAS9 technology and transplant these engineered cells to normal animals via skin grafting (Figure 1). Doxycycline inducible expression of GLP1 in grafted animals can reverse diet-induced obesity and diabetes [32]. Moreover, we also engineered a platform capable of delivering the BChE gene in reducing cocaine abuse. BChE is an endogenous enzyme that hydrolyzes its normal substrate acetylcholine [33]. BChE is secreted by hepatocytes and circulates in the blood. A computational designed version of BChE, the human BChE (hBChE), has significantly enhanced catalytic activity for metabolizing cocaine [34–36]. This designed enzyme can also decompose cocaethylene. We found that hBChE-expressing skin grafts could effectively metabolize cocaine in circulation at a fast rate and decrease DA levels in the NAc in the brain as quantified by using microdialysis followed by liquid chromatography–mass spectrometry (LC–MS), and protect mice from development of cocaine-taking and cocaine-induced drug-seeking, as measured by the conditioned place preference behavioral method, as well as cocaine overdose-related deaths [16,17], thus potentially providing a long-term solution for safeguarding against key features of cocaine abuse [18].

We recently utilized this gene delivery platform to examine its effectiveness in reducing alcohol reward and active ongoing consumption. We found that turning on GLP1 production from the grafted skin can effectively prevent development of alcohol-taking and reinstatement of alcohol-induced drug-seeking, as measure by CPP (Figure 1), as well as voluntary oral alcohol consumption in mouse models quantified by using a two-bottle choice behavioral paradigm (Figure 1, [15]. Moreover, skin-derived GLP1 can restrain alcohol-induced DA elevation in the brain mesolimbic DA system while having no effects on alcohol metabolism in the periphery [15]. This work expands the application of cutaneous gene delivery of GLP1 for treating diseased states beyond obesity and diabetes and adds valuable supportive evidence of targeting GLP1 system as a novel treatment for AUD. To explore the potential of this platform in managing polysubstance use, we developed an innovative co-culture and co-grafting procedure to co-express both GLP1 and hBChE. The simultaneous application of GLP1 and hBChE may bring higher efficiency in treating alcohol and cocaine co-abuse because hBChE can efficiently degrade both cocaine and cocaethylene thereby reducing their rewarding effects and toxicity, while GLP1 can reduce the reinforcing effect-induced by both alcohol and cocaine. We found that skin-derived GLP1 and hBChE could reduce alcohol and cocaine co-administration-induced acquisition of drug-induced CPP (Figure 1) and toxicity, suggesting the feasibility of using one skin stem cell-based gene delivery platform with a therapeutic gene cocktail delivered via skin grafts to address polysubstance abuse. Therapeutic gene cocktails have distinct advantages in the treatment of polysubstance abuse, because a single therapeutic agent may not effectively address all aversive effects produced by polysubstance abuse. They offer versatility and many opportunities involving different gene combinations in treating various polysubstance abuse.

The promising preclinical results for the use of the skin cell-based gene delivery platform to treat AUD and/or cocaine abuse in mice imply that this approach is long-lasting, highly efficient and minimally invasive with low maintenance. They offer hope that the approach may work in humans in the future because: 1) One platform can simultaneously address acquisition, reinstatement, ongoing use and overdose-related deaths. 2) The skin grafts will be autologous and the therapeutic GLP1 and hBChE genes are of human origin so the immune responses to the platform are expected to be low. 3) We have used keratinocytes isolated from human newborn foreskin in the *AAVS1* locus for targeting human GLP1 and hBChE genes in nude mice respectively and found strong GLP1 and hBChE production [17,32]. Grafted skins exhibited normal epidermal stratification, proliferation, and apoptosis *in vivo* with no tumorigenesis [17,32]. 4) hBChE has been tried in intramuscular injections in humans once weekly at a large amount of 300 mg and was shown to be well-tolerated and safe [37]. At least six GLP1 receptor agonists have been approved by the FDA to treat type II diabetes [38]. More recently, RYBELSUS^®^ (semaglutide, Novo Nordisk), became the first oral protein treatment approved for use in the US. Semaglutide is a protein that is chemically 96% identical to human GLP1. In human studies, after 26 weeks of use, 77% of those taking 14 mg once daily, patients do not experience significant side effects with about 1% weight loss while exhibiting significant improvement in type II diabetes. 5) Compared to all existing gene therapies, the skin cell-based gene therapy is much more affordable. Protocols for the isolation of skin stem cells, CRISPR targeting and preparation of genetically modified cultured epidermal autograft are well-established and routine technically. There are several commercial cultured epidermal autografts available (∼$1,000 per 100 cm^2^) [39] and the grafting procedures are relatively inexpensive [40]. The cutaneous gene therapy involves grafting of immunocompetent host, does not require lengthy hospitalizations, and the grafting procedure has been clinically used for treating burn wounds for decades.

Despite the optimism, there are challenges ahead when testing this approach in humans. 1) Will the platform have similar efficacy in humans as compared to that in mice? How can one provide sufficient quantities of therapeutic proteins to address different aspects of drug abuse and co-abuse? What are potential side-effects from the skin-derived therapeutic molecules? Despite of putting both hBChE and GLP1 genes under the control of a reversible regulator in doxycycline, how long can these genes be on without significantly interfering with endogenous acetylcholine and glucose homeostasis? What is the optimal ratio of proteins for treating drug abuse and co-abuse if a combination of therapeutic genes is used? What is the consequence of talking doxycycline long–term? 2) It is unclear how long the skin graft will last. Both GLP1 and hBChE skin grafts can last for at least 7 months in preclinical mouse studies and how much longer they remain effective in mice is still under investigation. Evidence from human studies indicates that skin grafts used in treating burn patients can last for a lifetime, and genetically modified skin patches for the treatment of junctional epidermolysis bullosa patients can remain stable for years [41], which implies the long-lasting potential of therapeutic skin grafts. 3) How do skin-derived GLP1 and hBChE impact the endogenous GLP1 and hBChE expression? Fully addressing these challenges will help adding a new option to treat drug abuse and co-abuse in the future.
